# Sexual Selection in Mosquitofish: Differences in the Use of Mating Cues Between Sexes

**DOI:** 10.3390/ani15101489

**Published:** 2025-05-21

**Authors:** Jiefei Wei, Bowen Feng, Chenglong Dong, Bojian Chen, Kai Liu

**Affiliations:** 1 Fisheries College, Jimei University, Xiamen 361021, China; jfwei1113@163.com; 2 State Key Laboratory of Mariculture Breeding, Fisheries College, Jimei University, Xiamen 361021, China; fengbowen0323@163.com; 3 College of Animal Science and Technology, Northwest A&F University, Yangling 712100, China; dongcl1996@nwafu.edu.cn; 4 Department of Biology, Queen’s University, Kingston, ON K7P 3N6, Canada; bjc18@queensu.ca

**Keywords:** mate choice, coercive mating system, mating cues, *Gambusia affinis*

## Abstract

Sexual selection is a major driver of speciation and evolution, with mate choice being a key component. Individuals assess mate quality by integrating various mating cues. The Western mosquitofish (*Gambusia affinis*), a species exhibiting pronounced sexual dimorphism in body size and secondary sexual traits, serves as an ideal model for studying mate choice. This study examines the impact of mating cues on mate choice in different sexes of *G. affinis* through a combination of morphological parameter database construction, computer-simulated animations, and dichotomous association preference tests. The results showed that male gonopodium status significantly affects female mate choice. Females exhibited a preference for males with resting-phase gonopodia, suggesting their aversion to forced copulation and sexual harassment in coercive mating systems. Furthermore, males preferred younger females, with this preference being positively correlated with male body size. This suggests that males are sensitive to sperm competition intensity and may base their choice on social rank. Geometric morphometric analysis and simulation experiments showed that males preferred females with larger gravid spots, regardless of age, suggesting that gravid spot size reflects female fecundity. Male preference for younger females with streamlined bodies and smaller abdomens was significant, but body size did not affect mate choice in general. Our findings highlight that female and male *G. affinis* employ different mate choice strategies, with females prioritizing male harassment avoidance and males considering multiple mating cues, not solely one dominant characteristic, in their mate choice decisions. These findings demonstrate that mate choice in *G. affinis* involves balancing conflicting preferences for traits associated with reduced harassment risk (e.g., resting-phase gonopodium in males) and those linked to reproductive potential (e.g., large gravid spot in females), highlighting the nuanced decision-making processes in both sexes.

## Simple Summary

Mate choice plays a crucial role in evolution, with individuals assessing potential mates based on various traits. The Western mosquitofish (*Gambusia affinis*), a sexually dimorphic species, provides an ideal model for studying these preferences. This study explored how mating cues influence mate choice in both sexes using morphological data, computer-simulated animations, and preference tests. Results showed that females preferred males with resting-phase gonopodia, likely to avoid forced copulation. Conversely, males preferred younger females, with preference strength increasing with male body size. Additionally, males favored females with larger gravid spots, indicating fecundity, but avoided larger, older females, possibly due to lower reproductive potential. These findings suggest that females prioritize avoiding harassment, while males consider multiple factors in mate selection, highlighting the complexity of mating strategies in *G. affinis*.

## 1. Introduction

Mate choice plays a critical role in shaping sexual selection, as individuals evaluate the quality of potential mates and make corresponding mating decisions based on the degree to which they weigh various sources of information. This process is also closely related to an individual’s reproductive fitness [1,2,3,4]. Several factors influence mate choice decisions, including morphological traits [5,6], behavioral characteristics including personality traits [7,8], and environmental factors [9,10,11]. Both sexes gather and analyze different types of information to make the most beneficial mating decisions. Therefore, the differences in the selection of mate selection cues between different genders have always been a hot topic in this field.

The purpose of animal mating is to pass on their own genetic material to their offspring by mating with a high-quality mate. In general, certain specific morphological characteristics of an individual can directly reflect certain physical conditions and survival abilities of the animal, such as the suitor’s resistance to parasites and diseases, predation ability, defense ability, and brooding ability [12,13]. Female guppies (*Poecilia reticulate*) prefer to mate with brightly colored males, especially males with orange spots on the sides of their bodies, indicating their physical health [14]. The morphological characteristics of females in many species are related to their fertility. For example, female two-spotted gobies (*Gobiusculus flavescens*) have bright orange-yellow abdomens. Male fish clearly prefer females with orange-yellow abdomens. The reason for this preference may be that the orange-yellow abdomen of female fish indicates their fertility [12]. Female *Heterandria formosa* prefers males with large body size [15], while another study did not find such a preference [16], suggesting that male size may not be the only factor affecting female mate selection. In addition, female Eastern mosquitofish (*Gambusia holbrooki*) generally prefer males with a medium mating frequency, which can avoid excessive sexual harassment from males and mating damage caused by forced mating, and obtain sufficient sperm to reproduce offspring [7]. Therefore, mate selection depends on the attractiveness of individuals of the same sex with different phenotypic characteristics to individuals of the opposite sex, including morphology, behavior, and the internal physiology and genes of the animal. This selection can be made directly for the animal itself or through the evaluation of the environmental conditions in which the animal is located. In short, in the process of mate choice, organisms integrate and evaluate various aspects of information about their potential mates to achieve the purpose of selecting the most suitable mate.

Due to reproductive investment differences, gamete disparity, and sexual selection pressures, females are generally choosier than males [3,17]. While many species exhibit female preferences for certain male traits, coercive mating systems deviate from this pattern. In such systems, males often use aggressive or coercive strategies, like sexual harassment or forced copulation, to secure mating opportunities and suppress female choice, shifting the evolutionary dynamics from mutual attraction to male-driven reproductive tactics [18]. These systems are common in species with high male density or intense competition for females, particularly in the Poeciliidae family, where males employ tactics such as trailing and gonopodium thrusting [19]. The gonopodium, a specialized sexually dimorphic anal fin, typically exhibits two positional phases in males. In the resting phase, the gonopodium remains laterally positioned adjacent to the abdominal region. During the active phase, the gonopodium shifts to a protruding forward position relative to the body. This is often followed by gonopodium movements, including lateral swings for courtship displays and thrusts for insemination [20,21,22,23]. In coercive systems, the gonopodium plays a crucial role as a specialized organ for sperm transfer, subject to strong sexual selection, with its morphology and length often indicating male health and competitiveness [24]. In the contexts where coercion is prevalent, males with relatively larger gonopodium may gain a reproductive advantage, as these traits enable successful copulation even when females resist [25]. However, females are not passive participants and may evolve adaptive traits, both morphological and behavioral, to resist male coercion, such as changes that make forced copulation more difficult [26]. While sexual coercion may increase male reproductive success, it often comes at significant costs to females, including time and energy expenditure, and sometimes even injury or death [27,28,29]. Therefore, the interaction between male mating cues (especially the gonopodium status) and the dynamics of coercive mating systems highlight the complex evolutionary pressures in fish mate choice.

Although studies on sexual selection have traditionally focused on male competition and female mate choice, increasing evidence suggests that males also exhibit mate choice behaviors, which are common in nature and play an important role in the evolution of mating systems [30,31,32]. In nature, females, especially, tend to prefer larger mates [2,33]. Larger individuals may have more advantages in survival and reproduction, such as better access to food resources, enhanced parenting and guarding abilities, and better protection from predators or male harassment [6,34,35]. Numerous studies have also shown that males prefer larger females [36], especially when female size is more strongly correlated with reproductive potential [6,37]. Theoretically, males aim to maximize mating success and prefer females with higher reproductive potential or those exhibiting traits associated with lower sperm competition intensity [38]. Research has shown that males of *H. formosa* prefer to mate with smaller (younger) females, as the risk and intensity of sperm competition are lower with these females, who are more likely to be virgins. The “virgin preference” is important in male mate choice behaviors. In poecilids, females can store sperm from multiple males, making sperm competition an important factor in mating systems [39]. Studies indicate that males tend to avoid sperm competition and prefer females who have recently mated with fewer or no other males [37,40]. Compared to relying on olfactory cues, the visual influence of morphological traits may be more important in determining female mating status [41]. In the life history of poecilids, male growth nearly ceases after sexual maturity, while females continue to grow throughout their lives, with increasing reproductive potential as they grow [6,20,42]. As females age, their morphological traits change dynamically, including increasing body size and changes in specific features such as gravid spots [20,43,44]. These mating cues are important indicators that males use in mate choice.

In poecilids, visual signals are an important means of obtaining external information [45] and play a significant role in mate choice, with morphological traits being one of the most important influencing factors. Poecilids, due to their marked sexual dimorphism, are ideal model organisms for mate choice studies. It has been reported that male poecilids exhibit clear mate preferences [37,46], suggesting that their sexual selection is driven by both sexes. While numerous studies have focused on mate choice related to morphological traits in poecilids, research on the effects of gonopodium status and age-related mating cues remains limited. Western mosquitofish (*Gambusia affinis*), a representative species of Poeciliidae, follows a promiscuous mating system with no parental care or territorial behavior by males [47]. Given that male size ceases to increase after sexual maturity [21,48], males maturing earlier tend to be smaller, while those maturing later are relatively larger. This study focuses on *G. affinis* to explore two key questions through binary mate choice experiments: (1) How does the status of the male gonopodium affect female mate choice? (2) How does female age affect male mate choice? This research contributes to understanding “bidirectional mate choice” and provides a theoretical basis for understanding the complex and variable mating strategies in natural populations.

## 2. Materials and Methods

### 2.1. Origin and Maintenance of Test Fish

Wild-caught Western mosquitofish *G. affinis* were collected from Ankang City, Shaanxi Province (120°15.58′ N, 30°27.70′ E), all of which were sexually mature individuals. The fish were maintained in several aerated and filtered 200 L aquaria equipped with aquatic plants, driftwood, and rocks for shelter. They were fed twice a day (at 10:00 a.m. and 4:00 p.m.) ad libitum amounts of bloodworms, brine shrimp, and commercial fish flakes. The stocking density in the aquariums was 40 fish per tank, with a sex ratio of approximately 1:1. The water temperature in the tanks was maintained at 25 ± 0.1 °C, with a photoperiod of 12 h per day. Half of the water was replaced every two weeks, with both the rearing and experimental water being sourced from dichlorinated tap water. All animals were allowed to acclimate under laboratory conditions for 2 weeks before experimentation.

### 2.2. Test 1- Influence of Male Gonopidium Status on Mate Choice in Females

#### 2.2.1. Creation of Male *G. affinis* Animations

According to the fish image database created by Chen et al. [49] for *G. affinis*, 20 images were randomly selected from a total of 24 male fish images. These images were animated using Macromedia Flash 8.0 and converted into .flv files (resolution: 1024 × 768; 60 frames/s). The final set of animations consisted of virtual male fish pairs, where each pair showed different gonopodium phase (e.g., active/resting phase) to reflect relative exposure. The only difference between the two was the gonopodium status, and other morphological traits, such as body length (22.30 mm), body color, swimming speed (2.63 cm/s), and background, remained consistent across all pairs. Each animation displayed a virtual male fish swimming horizontally from the left to the right side of the screen against the light gray background, and the male performed a back-and-forth swimming motion. Before changing direction, the virtual male would swim an additional body length distance off-screen, out of the view of the experimental fish, to simulate an invisible turn. To avoid pseudo-replication, the study utilized the 20 high-resolution images of female *G. affinis* as templates to create 20 distinct animation pairs (Supplementary Materials, Figure S1). Additionally, we maintained proportional scaling using Adobe Photoshop CC 2022 (23.0)’s “Content-Aware Scale” tool to avoid distortion.

#### 2.2.2. Female Mate Choice Test

Prior to the experiment, male fish were placed in isolated 1.5 L transparent plastic bottles for 24 h. The isolation tanks were covered with black plastic on three sides and the bottom, and the plastic bottles were suspended inside the isolation tanks, each with small holes for water and gas exchange. The water temperature in the isolation tanks was maintained at 25 ± 0.1 °C, with a 12 h light/dark cycle and sufficient oxygenation. A dichotomous association preference test was conducted following the setup described by Chen et al. [49]. The standard length (SL) of each fish was measured with a precision of 0.1 mm at the end of the experiment. Water in the experimental tank was replaced after every four trials to maintain water quality. Finally, the total time spent in each preference zone was calculated, and the strength of mate preference (SOP) was determined. A total of 33 female fish (Mean ± SE, SL: 30.42 ± 4.66 mm) were tested in the experiment.

### 2.3. Test 2- Influence of Female Age on Mate Choice in Males

#### 2.3.1. Acquisition of High-Resolution Images of Young and Old Female Fish

This study involved the collection of high-resolution images of female fish captured at different time points to create computer animations. The “older” females were captured in Ankang City and were kept in the laboratory for at least one year before the images were taken. The “younger” females were subadult females, and they reached sexual maturity within one month of being kept in the laboratory. Studies have shown that the natural lifespan of wild *G. affinis* typically does not exceed 12–15 months, although individuals raised under controlled conditions can live up to 18 months [50,51]. Thus, the “older” females were at least one year old, while the “younger” females were no more than 0.5 years old, with a minimum age difference of six months between the two groups. Following the methodology of Chen et al. [49], a total of 40 high-resolution images of wild-caught and laboratory-raised female *G. affinis* were obtained (Mean ± SE, SL_Young female_: 28.15 ± 4.57 mm, *n* = 20; SL_Old female_: 37.30 ± 2.84 mm, *n* = 20).

#### 2.3.2. Quantify the Relative Gravid Spot Area of Young and Old Female Fish

**Figure 1:**
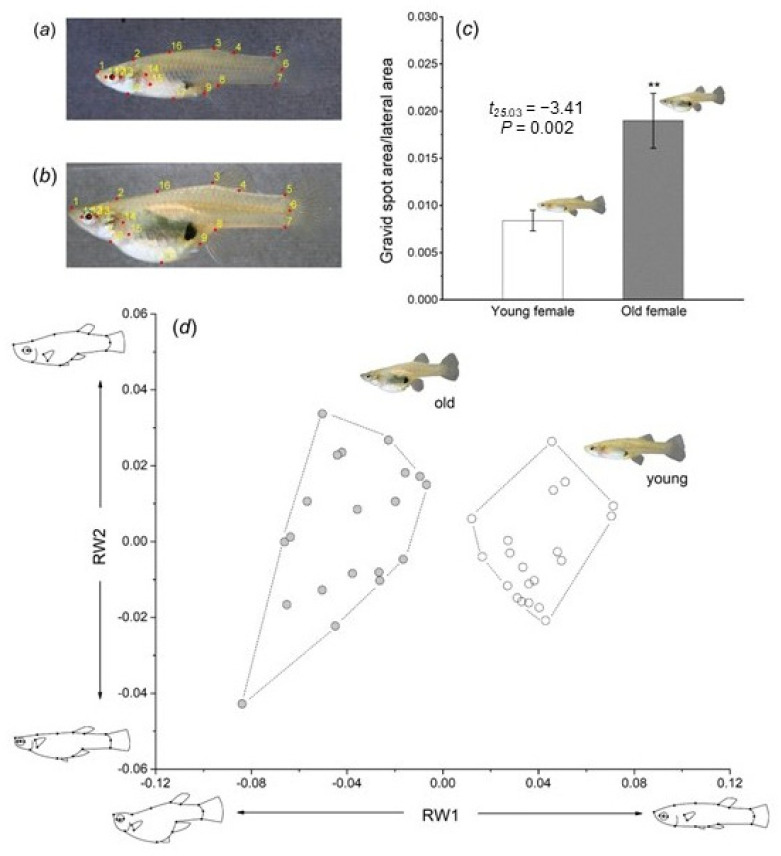
Morphological landmarks, as well as differences in body shape and coloration between young and old female *G. affinis*. Dots and numbers indicate the 17 landmarks used for morphometric analyses in (**a**) young and (**b**) old females. (**c**) Ratio of ‘gravid spot area/lateral area’ in young and old females with the results of an independent-samples *t*-test. (**d**) Body shape variation along RW1 and RW2. ** refers *p*<0.05.

**Figure 2:**
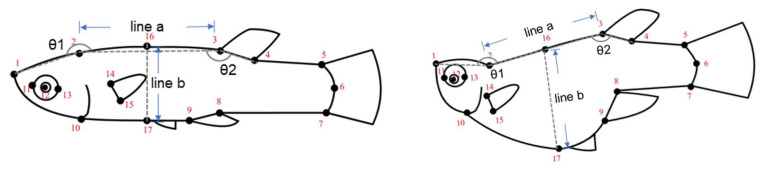
Spinal and abdominal shape manipulation reference for computer-animated female stimuli.

Using the software Morpho J 1.08.02., the areas of the gravid spot and the lateral body surface of the *G. affinis* were measured from 40 high-resolution photographs [52], and the ratio between the two areas was calculated. Comparison revealed that the relative gravid spot area of young females (gravid spot area/lateral body surface: Mean ± SE = 0.84 ± 0.11%) was significantly smaller than that of older females (1.87 ± 0.29%, independent sample *t*-test, *t*_25.03_ = −3.41, *p* = 0.002, Figure 1c). This indicates that young females have relatively smaller gravid spots, while older females have relatively larger gravid spots, with a significant difference between the two groups. The geometric morphometric analysis followed three steps [53]: (1) the photos were converted to tps format using tpsUtil 1.82 software, and 17 landmark points were digitized with tpsDig 2.05 software. These landmarks provided a complete representation of the lateral body contour of the female fish (Figure 1a,b); (2) a Procrustes fit analysis was performed using Morpho J, and data reduction was applied during shape extraction; and (3) 10 principal components (RW) were extracted from the shape features of the females, accounting for 94.03% of the total shape variation (Table 1), with the first two principal components visualized (total shape variation accumulated to 69.36%; Table 1). Our results showed significant differences in the spine and abdominal morphology between young and older females. Specifically, young females exhibited a streamlined spine and smaller abdominal area, while older females had a “Z”-shaped spine and larger abdominal area (Figure 1d).

#### 2.3.3. Creation of Female *G. affinis* Animations

During the creation of computer-simulated animations, the swimming speed of all female fish (2.71 cm/s) and the background (light gray) were kept consistent. Except for in the experiments described in Section 2.3.4, the standard body length of the virtual female fish remained constant across all experimental conditions (Mean ± SD, SL: 29.1 ± 4.67 mm). Apart from in the experiments described in Section 2.3.4, the animations displayed two images of the same individual from both sides, with differences in specific morphological traits. Each animation depicted a virtual female fish swimming horizontally from the left side of the light gray background screen to the right, with a return movement. Before changing the swimming direction, the virtual female fish executed an invisible turn equivalent to one body length. To avoid pseudoreplication, we used 40 high-resolution photographs of female *G. affinis* as templates, generating at least 20 pairs of different animations for each experimental group. The images were processed using Macromedia Flash 8.0 and converted into .flv files (resolution: 1024 × 768; 60 frames/s).

#### 2.3.4. Male Preference for Females of Different Ages

The images of the 40 female fish were randomly paired in pairs to create animations representing both young and aged morphologies. Each animation pair consisted of a virtual young and a virtual old female fish (Supplementary Materials, Figure S2). The standard body length of all virtual female fish was adjusted to 22.30 mm.

#### 2.3.5. Male Preference for Females with Different Relative Areas of Gravid Spots

The ratio of the gravid spot area to the body side area indicates that young females have relatively smaller gravid spots. Therefore, to test whether males can distinguish between young and old females based on the relative area of the gravid spot, this study created two sets of animated pairs (“small” and “large” gravid spot area/body side area ratios) using images of young and old females, respectively. The “small” ratio was defined as the average ratio of gravid spot area to body side area in young females, while the “large” ratio was defined as the average ratio in old females. Additionally, Adobe Photoshop CS5 was used to adjust the gravid spot area in each image to a predefined value, thereby creating the corresponding animations.

#### 2.3.6. Male Preference for Females with Different Morphological Traits (Spine and Abdomen Morphology)

The results of the geometric morphometric analysis indicate that the morphological differences between young and old females are primarily related to the shape of the spine and abdomen. Based on these two key characteristics, a full-factorial design binary mate choice experiment was conducted (six experimental groups: a, b, c, d, e, and f), with 20 pairs of animated images produced for each group. The female fish images used in the animations were derived from young and old female fish. To adjust the morphology of the spine and abdomen of the female fish, Adobe Photoshop CS5 was employed with three parameters as anchor points: θ1, θ2, and the ratio of b-line/a-line (R) (Figure 2). θ1 is the angle between the head and back of the female fish, formed by the connection of landmark points 1, 2, and 2, 3 (mean values: θ1_young_ = 197.2°, θ1_old_ = 162.4°). θ2 represents the angle between the back and tail, formed by the connection of landmark points 2, 3, and 3, 4 (mean values: θ2_young_ = 163.4°, θ2_old_ = 151.1°). The combination of θ1 and θ2 represents the spinal morphology of females of different ages. The a-line is the distance between landmark points 2 and 3, while the b-line is the distance between landmark points 16 and 17. The R-value can represent the abdominal morphology of young (Mean R_young_ = 0.53) or old (Mean R_old_ = 0.86) females.

In experiments a–c, the control animations for each pair were derived from images of young female fish, while the experimental animations were generated by modifying the images of young female fish to exhibit the morphological characteristics of older female fish. These modifications included changes to spinal shape (experiment a: using Adobe Photoshop CS5 to adjust the body angles θ1_young_ and θ2_young_ to resemble θ1old and θ2old of older female fish), spinal shape combined with abdominal morphology (experiment b: adjusting θ1_young_, θ2_young_ and R_young_ to θ1_old_, θ2_old_ and R_old_), and abdominal shape (experiment c: adjusting R_young_ to R_old_).

In experiments d–f, the animation creation method was similar to that of experiments a–c, with the distinction that in these cases, the control animations were based on images of older female fish, and the experimental animations were processed to exhibit the morphological traits of younger female fish. Specifically, experiment d involved changes to the spinal shape, experiment e combined changes to both spinal and abdominal morphology, and experiment f focused on changes to abdominal shape.

### 2.4. Statistical Analyses

All statistical analyses in this study were performed using SPSS 19. First, the strength of the female fish mate preference for males with either a continuously active/resting-phase gonopodium was measured, referred to as the mate preference strength (SOP), calculated as follows: SOP = (time spent in the preference area for the virtual male with the continuously active-phase gonopodium) − (time spent in the preference area for the virtual male with the resting-phase gonopodium)/total time spent in both preference areas.

Thus, the SOP value ranges from 1 (indicating a preference for males with continuously active-phase gonopodia) to −1 (indicating a preference for males with resting-phase gonopodia). A one-sample *t*-test was used to compare the SOP values to a hypothesized constant (SOP = 0), testing the overall preference of the females and the experimental conditions. Subsequently, a general linear model (GLM) was conducted with SOP as the dependent variable, including animation number as a random factor and female standard body length as a covariate.

Then, in the experiments investigating male fish preference for female fish of different ages, male preference for female fish with varying relative areas of pregnancy spots, and male preference for female fish with different morphological traits (spinal and abdominal morphology), raw data were used for statistical analysis. Unless otherwise specified, all descriptive statistics are expressed as Mean ± SE.

To determine the strength of mate preference (SOP) for young female fish (characterized by traits such as gravid spot size, spinal, and abdominal morphology), we used the following formula: SOP = (time spent near the preference zone of young trait − time spent near the preference zone of old trait)/total time spent in both preference zones. In the gravid spot and body shape tests, body size was not standardized due to image manipulation constraints. We therefore used SOP value adjustments to account for size bias.

To analyze the overall preference of each group and the experimental results, a one-sample *t*-test was used to analyze the overall SOP values of the experimental fish. A general linear model (GLM) analysis was then performed with SOP as the dependent variable. In the experiments on male preference for female fish of different ages, male preference for female fish with varying relative areas of pregnancy spots, and male preference for female fish with different morphological traits (spinal and abdominal morphology), when processing the three sets of experimental data, animation type (animation type: animations of young or old female fish) was set as a fixed factor, and the standard body length of the experimental male fish (male body size) was included as a covariate in the GLM analysis. In the experiment on male preference for female fish with different morphological traits (spinal and abdominal morphology), when conducting the GLM analysis, animation type, spinal morphology, and abdominal morphology were treated as fixed factors, and the standard body length of the experimental male fish (male body size) was treated as a covariate. A subset analysis was then performed on the data from the young and old female fish animations, excluding the animation type factor. Initially, animation ID was treated as a random factor in the GLM analysis. However, since the *p*-value for animation ID was non-significant (*p* > 0.19) in all analyses, it was excluded from the final model.

## 3. Results

### 3.1. Female Preference for Males with Different Gonopodium Status

**Figure 3:**
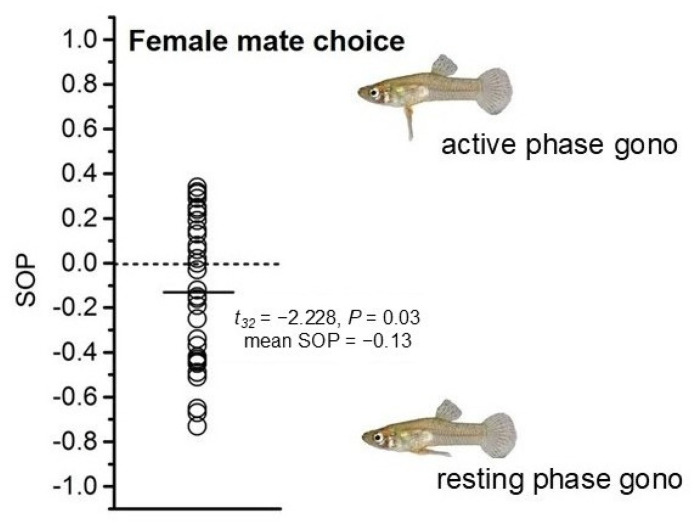
Distribution of individual strength of preference (SOP) values derived from dichotomous association: female choice for male with active-phase gonopodium.

A general linear model revealed no significant differences in the random variable “Animation ID” and the covariate “Standard Length (SL) of female fish” (Table 2). In assessing the female fish’s preference for males with different gonopodial status, it was found that the time spent near males with resting-phase gonopodia was significantly longer than the time spent near males with active-phase gonopodia. By calculating the strength of mate preference and performing a one-sample *t*-test, it was determined that female fish showed a significant aversion to males with persistently active-phase gonopodia (mean SOP = −0.13, *t*_32_ = −2.228, *p* = 0.03, Figure 3).

### 3.2. Male Preference for Females of Different Ages

**Figure 4:**
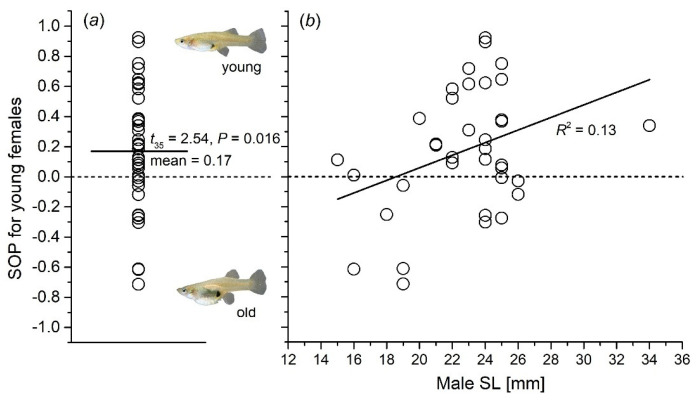
Male mating preference for young female phenotypes in *G. affinis*. (**a**) Distribution of individual strength of preference (SOP) values derived from dichotomous association: male choice for young and old females; (**b**) Relationships between male body size (SL) and SOP for young females.

Compared to the time spent near the animations of older female fish, male fish spent significantly more time near the animations of younger female fish. A one-sample *t*-test on the corresponding SOP values revealed a significant difference (*t*_35_ = 2.54, *p* = 0.016; Figure 4a). A general linear model indicated that male body size significantly influenced male preference for younger female fish (*F*_1, 34_ = 4.99, *p* = 0.032), with a positive relation between mate preference and body size (*R*^2^ = 0.13; Figure 4b).

### 3.3. Male Preference for Females with Different Relative Areas of Gravid Spots

**Figure 5:**
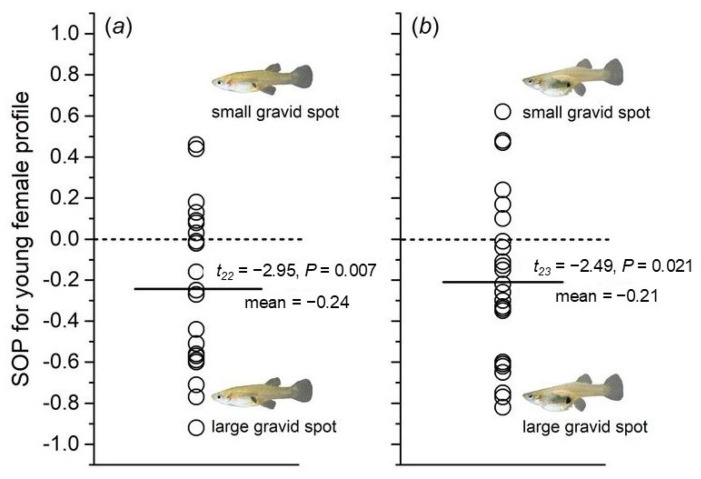
Male mate choice based on gravid spot size. Computer animations showed stimulus females with a large or a small gravid spot and were created using images of (**a**) young and (**b**) old females.

The results were contrary to the prediction, as males spent more time near the animation of females with larger gravid spot areas (Figure 5). When using the young female fish image as a template to create computer animations, the experimental results showed that males spent significantly more time near the animation of females with larger gravid spot areas compared to those with smaller gravid spot areas. A one-sample *t*-test of the corresponding SOP values revealed a significant difference (*t*_22_ = −2.95, *p* = 0.007; Figure 5a). When using the older female fish image as a template, the results showed that males spent significantly more time near the animation of females with larger gravid spot areas than near those with smaller gravid spot areas. A one-sample *t*-test of the corresponding SOP values showed a significant difference (*t*_23_ = −2.49, *p* = 0.021; Figure 5b). A general linear model showed it was neither the animation model nor the body size of the experimental fish had a significant effect on the corresponding SOP values (Table 3).

### 3.4. Male Preference for Females with Different Morphological Traits (Spine and Abdomen Morphology)

**Figure 6:**
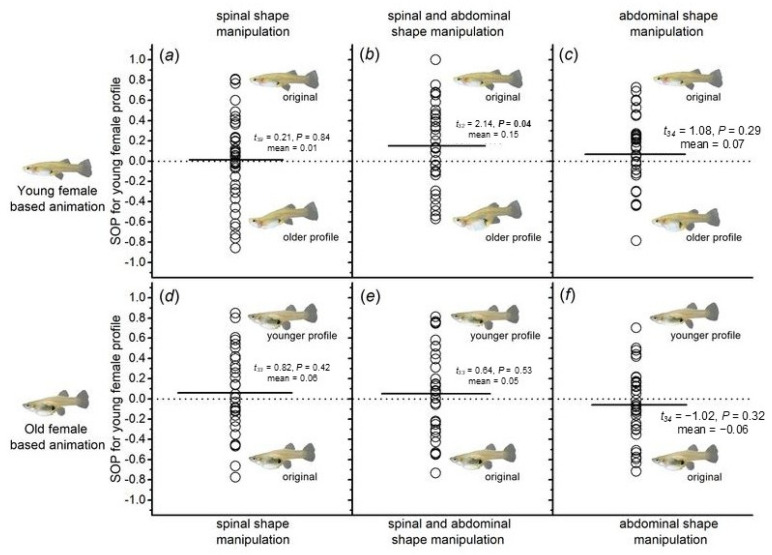
Distribution of individual strength of preference (SOP) values derived from dichotomous association preference tests involving (virtual) spinal and abdominal shape manipulations in stimulus females.

In six experimental groups, significant differences in SOP values were observed only when the animation templates were derived from young female fish images, and both spinal and abdominal morphologies were altered to simulate those of older female fish. In this case, the time spent by male fish in the preference area of the young female fish animation was significantly longer than the time spent in the preference area of the virtual older female fish animation. The corresponding SOP values were calculated, and a one-sample *t*-test revealed significant differences (*t*_32_ = 2.14, *p* = 0.04; Figure 6b). In contrast, the results from the other five experimental groups (Figure 6a,c–f) showed no significant preference by male fish for any of the female fish animations. Analysis using a general linear model indicated that none of the factors—animation type (whether the animation was based on young or old female fish), spinal shape, abdominal morphology, or the body size of the experimental fish—nor their interactions, had a significant effect on the results (Table 4).

## 4. Discussion

Our study utilized computer-generated animations to simulate male *G. affinis* with different reproductive traits and conducted mate preference experiments to assess female responses. The male fish were kept consistent in various traits, including body size, swimming behavior (such as swimming speed and direction), body coloration, and reproductive fin size. The only variation was in the status of the gonopodia, which were presented in either an active or a resting phase form. The results revealed that female fish significantly preferred males with resting-phase gonopodia, showing a marked aversion to males whose fins remained constantly in an active phase status. This behavior aligns with previous findings in species where males invest relatively little in offspring, opting instead to increase their reproductive success through high-frequency mating. By maximizing copulation attempts, males aim to produce more offspring, often at the expense of female well-being [2]. In many sexually dimorphic species, males engage in sexual harassment as a strategy to force copulation and secure fertilization [54,55,56]. Male *G. affinis* often engage in sexual harassment of females [57] and force copulations to achieve fertilization. When males sexually harass females, the females’ reproductive investment significantly increases, potentially leading to injuries such as genital damage [58]. As a result, females tend to resist most of the males’ mating attempts. During the initial mating attempt, males swing their gonopodia to the side and rear of the female to insert their fins into the female’s genital opening for ejaculation, thus raising the fins into an active phase status [20]. Female rejection of males with consistently active-phase gonopodia may be a response to their aversion to forced copulations and sexual harassment. Frequent mating can cause significant harm to females, such as genital injury [58] and reduced foraging efficiency [29,59]. Moreover, sexual harassment increases an individual’s exposure to predators, thereby elevating the risk of predation [60], which reduces both survival and reproductive fitness. Notably, the lack of response to a cue does not imply a lack of perception but may indicate differing decision thresholds.

In the animal kingdom, mate choice is often seen as a process where females exert dominant influence, especially when it comes to the selection of high-quality mates. However, our study showed males can also exhibit a highly selective preference in their mate choices. Actually, male mate choice does not contradict general patterns but illustrates sex-specific decision strategies. Specifically, we found that male *G. affinis* showed a strong preference for younger females, a preference that is closely linked to the male’s body size. The strength of this mate preference (SOP) was positively correlated with the male’s body size, suggesting that larger males might be more capable of distinguishing subtle differences in female age and body traits, which could signal higher reproductive potential. This finding is noteworthy because it implies that males do not merely choose mates based on physical attributes like size or coloration, but also on age-related traits that may be indicative of fertility or overall health [61]. The preference for younger females may be related to the fact that younger individuals are typically at their peak reproductive age, which could lead to more successful and healthier offspring [62,63]. Additionally, it is important to consider the role of sperm competition, a well-documented phenomenon in many species. Virgin females, due to the lower intensity of sperm competition within their reproductive tract, may offer a more favorable environment for sperm fertilization. This could explain why males might preferentially choose younger females, as they are likely to be virgins and therefore experience less sperm competition, ensuring a higher likelihood of successful fertilization. As virgin females tend to experience lower levels of sperm competition, many species exhibit a preference for virgin mates [64]. For example, males from the species *H. formosa* prefer smaller females, possibly because they offer a more favorable environment for sperm success due to reduced sperm competition [16]. Hence, male *G. affinis* may not only be selecting mates based on direct physical or age-related traits but also strategically considering the potential benefits of reduced sperm competition. This selective behavior could be an adaptive strategy to maximize reproductive success and ensure that the male’s genetic material has a better chance of fertilizing the female’s eggs.

Morphological analysis of young and old female fish revealed significant differences in the size of the gravid spot (color pattern), as well as spine and abdominal morphology (morphological traits). When the relative area of the gravid spot was used as a variable, males consistently preferred females with a larger relative area of the gravid spot, with female age becoming a potential influencing factor. In most fish species, the size of the gravid spot is used as a marker to identify the developmental stage of females [44]. Studies have shown that the gravid spot undergoes dynamic changes during different developmental stages [20,43,44]. Research on the life history of certain mosquito fish species indicates that the size and pigment intensity of the gravid spot may be linked to the female’s reproductive cycle—spots grow larger and become more intensely colored with age [20,65]. While body size in female fish represents their reproductive capacity, the relative area of the gravid spot may provide a more intuitive indication of their fertility. Therefore, males consistently prefer females with a larger gravid spot, regardless of the female’s age. When spine and abdominal morphology were used as variables in a full-factorial design and binary mate choice experiments were conducted, it was found that males showed a significant preference for females with “young abdominal and spine morphological traits”. The apparent contradiction between this preference and the males’ strong preference for females with a larger gravid spot (an indicator of older age) suggests that male *G. affinis* do not rely on a single morphological characteristic to select potential mates. In studies on the impact of body size on male mate choice, males showed no significant preference for females with larger body sizes, whether the females were young or old. This result contrasts with the earlier finding of males’ preference for younger females, suggesting that body size is a potential preference trait but is overshadowed by age. Research has shown that with age, female body length increases, along with reproductive capacity [20]. However, since males did not prefer larger females, it suggests that body size does not intuitively reflect a female’s reproductive potential. Additionally, as female body size increases with age, older females may have lower overall fitness.

In natural environments, mate choice in many species does not rely on a single trait but rather on the integration of multiple cues. This strategy of multi-cue integration is thought to enhance the flexibility and adaptability of decision making, particularly in contexts where signals are variable or environmental conditions are unpredictable [66]. For *G. affinis*, factors such as body size, gravid spot size, age, and harassment risk may all serve as parallel sources of information with differing weights. Males and females are likely to integrate and balance these cues when making mate choices, leading to individual differences in preference patterns [4]. Therefore, we interpret the subtle deviations in SOP values observed in our study as reflecting a complex cognitive evaluation process in *G. affinis*.

## 5. Conclusions

In summary, we found that during the mate choice process of *G. affinis*, females exhibited a strong aversion to males with continuously active phase genital fins, indicating a clear rejection of forced mating and male sexual harassment. In the male mate choice process, males preferred to mate with younger females, possibly due to the lower intensity of sperm competition associated with younger females. Additionally, larger males showed a stronger rejection of older females, likely because larger males have higher social status and more mating opportunities, making them more “choosy” in mate choice compared to smaller males. Furthermore, male *G. affinis* showed a significant preference for females with larger gravid spots (an indicator of older morphological traits) and those with streamlined bodies and smaller abdomens (indicating younger morphological traits). This suggests that no single mating cue dominates male mate choice. Future research could explore the plasticity of mate choice in this species during its invasive expansion, particularly under extreme environmental changes, and investigate the evolutionary patterns of genital fin morphology and behavioral traits in sexual selection, revealing the adaptive mechanisms behind the reproductive behaviors of this species.

## Figures and Tables

**Table 1:** Results of a principal component analysis on morphological variation.

Relative Warps (RW)	Eigen Value	Variance Explained (%)
RW1	1.91 × 10^−3^	60.90
RW2	2.65 × 10^−4^	8.46
RW3	2.34 × 10^−4^	7.46
RW4	1.13 × 10^−4^	3.59
RW5	1.06 × 10^−4^	3.39
RW6	8.74 × 10^−5^	2.79
RW7	7.95 × 10^−5^	2.54
RW8	5.90 × 10^−5^	1.88
RW9	5.51 × 10^−5^	1.76
RW10	3.95 × 10^−5^	1.26

**Table 2:** Female preference for males with active-phase gonopodia.

Source	*df*	*F*	*p*	Wilks’ Partial *η*_p_^2^
Animation ID	20	1.37	0.28	0.66
SL	1	0.13	0.72	0.009
Error	30			

**Table 3:** Results of general linear model (GLM) using SOP values for gravid spot size as dependent variables; Animation pairs were derived from images of young or old females.

Factor	*df*	*F*	*p*	Wilks’ Partial *η*_p_^2^
Animation type	0	-	-	-
Animation ID	18	0.92	0.57	0.40
Male body size	1	0.55	0.47	0.022
Animation type × male body size	1	4.21	0.051	0.14
Error	25			

**Table 4:** Results of general linear model (GLM) using SOP values for spinal and abdominal shape as dependent variables; Animation pairs were derived from images of young or old females.

Source	*df*	*F*	*p*	Wilks’ Partial *η*_p_^2^
(a) Both animation types combined				
Animation type	1	0.20	0.66	0.001
Spinal shape	1	0.05	0.83	0.0002
Abdominal shape	1	1.38	0.24	0.01
Male body size	1	1.88	0.17	0.01
Error	206			
(b) Animations derived from young females				
Spinal shape	1	0.81	0.37	0.01
Abdominal shape	1	2.21	0.14	0.02
Male body size	1	0.11	0.74	0.001
Error	104			
(c) Animations derived from old females				
Spinal shape	1	1.78	0.19	0.02
Abdominal shape	1	0.03	0.86	0.0003
Male body size	1	3.05	0.084	0.03
Error	99			

## Data Availability

The original contributions presented in the study are included in the article; further inquiries can be directed to the corresponding author.

## Supplementary Materials

The following supporting information can be downloaded at: https://www.mdpi.com/article/10.3390/ani15101489/s1, Figure S1: Images of males (M1–M20) used to generate computer animations; Figure S2: Images of females (Pair1–Pair20) used to generate computer animations.
