# Supplementary material for: Sexual Selection in Mosquitofish: Differences in the Use of Mating Cues Between Sexes
Source: Animals (Basel). 2025 May 21;15(10):1489. doi: 10.3390/ani15101489 (PMC12108260; doi:10.3390/ani15101489)
Supplement: Supplementary file 1 [file animals-15-01489-s001.zip › animals-3581140-supplementary.pdf]

animals

Supplementary Materials

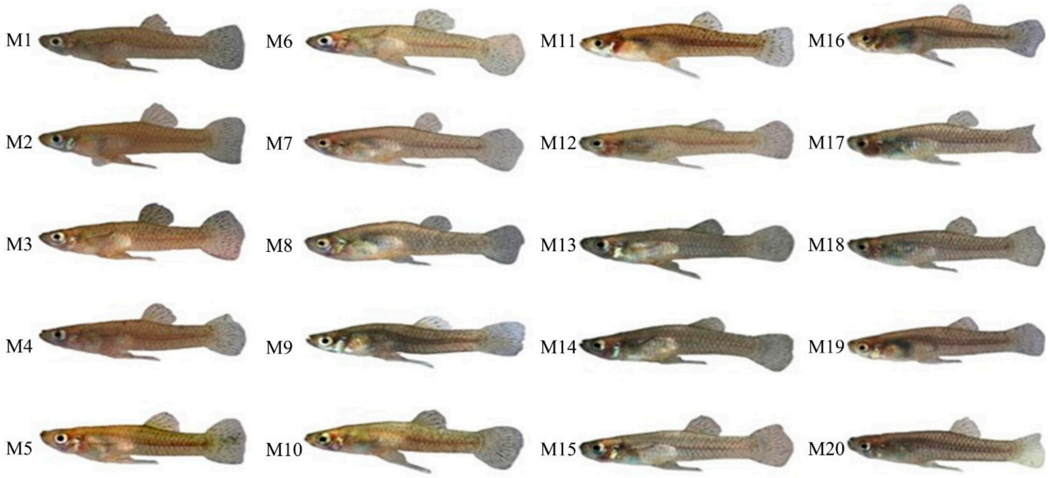

Figure S1. Images of males (M1–M20) used to generate computer animations.

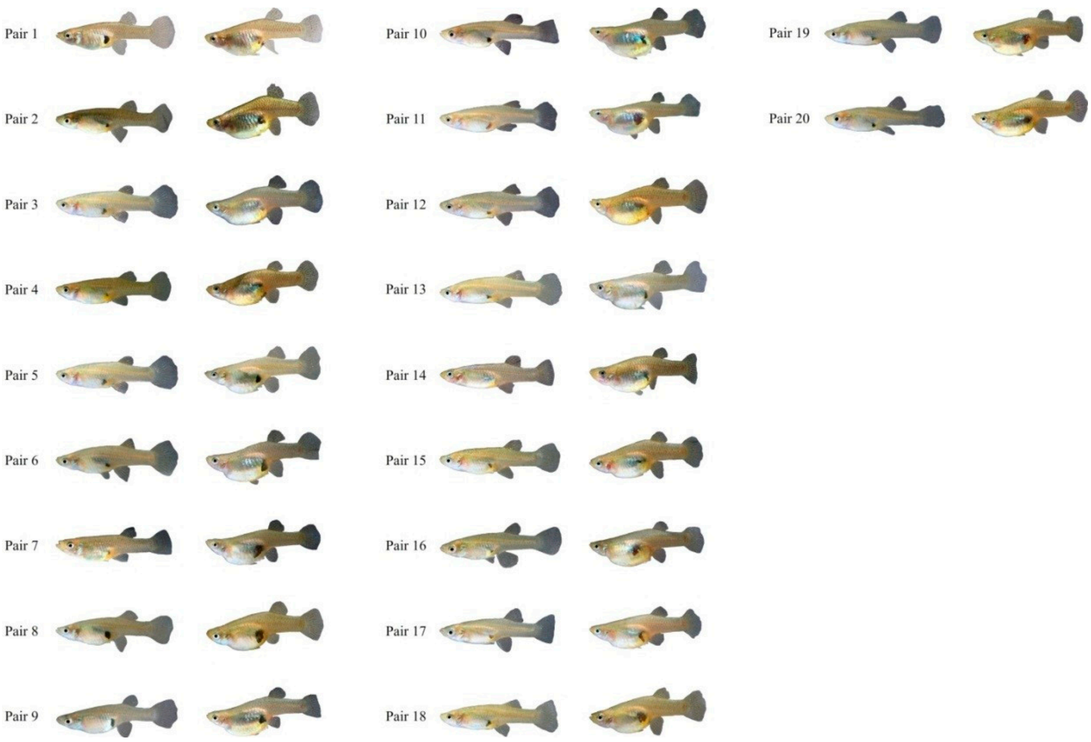

Figure S2. Images of females (Pair1–Pair20) used to generate computer animations.
